# Neural Processing of Facial Identity and Emotion in Infants at High-Risk for Autism Spectrum Disorders

**DOI:** 10.3389/fnhum.2013.00089

**Published:** 2013-04-09

**Authors:** Sharon E. Fox, Jennifer B. Wagner, Christine L. Shrock, Helen Tager-Flusberg, Charles A. Nelson

**Affiliations:** ^1^Massachusetts Institute of TechnologyCambridge, MA, USA; ^2^Children’s Hospital BostonBoston, MA, USA; ^3^College of Staten Island, The City University of New YorkNew York, NY, USA; ^4^Harvard UniversityCambridge, MA, USA; ^5^Boston UniversityBoston, MA, USA

**Keywords:** autism, fNIRS, face processing, near-infrared spectroscopy, cognitive development

## Abstract

Deficits in face processing and social impairment are core characteristics of autism spectrum disorder. The present work examined 7-month-old infants at high-risk for developing autism and typically developing controls at low-risk, using a face perception task designed to differentiate between the effects of face identity and facial emotions on neural response using functional Near-Infrared Spectroscopy. In addition, we employed independent component analysis, as well as a novel method of condition-related component selection and classification to identify group differences in hemodynamic waveforms and response distributions associated with face and emotion processing. The results indicate similarities of waveforms, but differences in the magnitude, spatial distribution, and timing of responses between groups. These early differences in local cortical regions and the hemodynamic response may, in turn, contribute to differences in patterns of functional connectivity.

## Introduction

Deficits in face processing are thought to be a characteristic of autism, though the nature and development of these deficits has yet to be fully determined. Typically developing infants acquire the ability to recognize their mothers’ face within weeks after birth (de Haan and Nelson, 1997; Pascalis et al., 1998). In this same time frame, infants also learn to recognize basic facial emotions. Several groups have employed neuroimaging methods to understand the ontogeny of facial identity and emotion processing (Taga et al., 2003; Otsuka et al., 2007; Minagawa-Kawai et al., 2009; Nakato et al., 2009, 2011; Honda et al., 2010). Studies involving functional Near-Infrared Spectroscopy (fNIRS) have shown effects of face processing to be dominant in the lateral right hemisphere during early development (Otsuka et al., 2007; Nakato et al., 2009; Honda et al., 2010). Minagawa-Kawai et al. (2009) found differences in fNIRS response patterns between viewing of emotional mother and stranger video conditions in 11-month-old infants using only four channels placed over orbitofrontal cortex (OFC), suggesting that this region of the brain is involved in distinguishing familiar from unfamiliar faces. Additional studies involving fNIRS, as well as other imaging modalities (e.g., fMRI), have also revealed the role of OFC and neighboring frontal cortex in processing positive affect (Grossmann et al., 2010; Blasi et al., 2011; Goodkind et al., 2011; Ito et al., 2011; Volkow et al., 2011).

**Figure 1:**
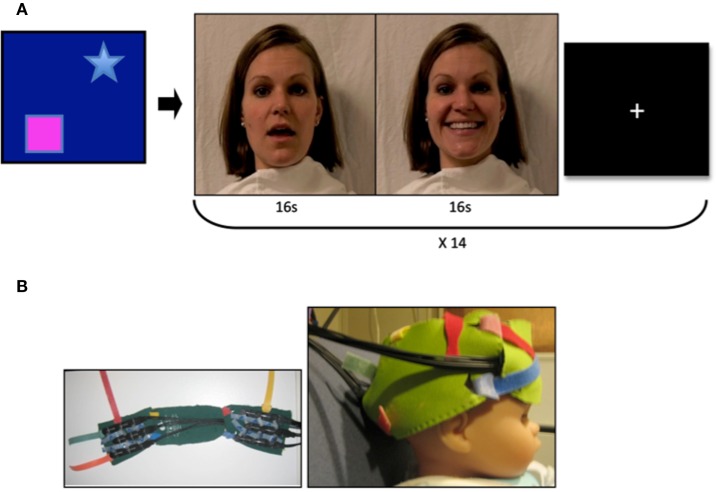
**Video stimuli and probes**. **(A)** Mothers of infant participants were instructed to answer a series of questions with both a neutral and smiling expression. Sound was removed from the videos, and 16 video clips of each emotion were combined to achieve a 32 s stimulus with transition from neutral to smiling expression. Stranger videos were selected for similarity in appearance to the mother. A visual baseline of moving objects was used to direct infant attention to the screen at the beginning of the experimental session, and a fixation cross was present between stimulus presentation. **(B)** Two chevron arrays of probes placed into a custom designed neoprene cap. Probes were placed in the frontal and right lateral panels, and adjusted to proper fit and location with the colored Velcro tabs.

Given the robust nature of face processing deficits in individuals with autism, one might speculate that such deficits may predate onset of the disorder itself, and thus be present, as an endophenotype, in infants at high familial risk for autism. An fMRI study of unaffected brothers of individuals with ASD performing a visual integration task revealed that they share a pattern of atypical frontal hypoactivation with their affected siblings (Belmonte et al., 2010). In addition, similar atypical structural differences in the white matter of frontal and temporal regions involved in social cognition have been reported in both children with autism and their unaffected siblings (Barnea-Goraly et al., 2010). These findings lend support to the hypothesis of a “high-risk” endophenotype, which may be present in individuals with autism, as well as their unaffected siblings. Detection of these endophenotypes early in infancy could potentially provide risk stratification, as well as information about compensatory mechanisms or protective factors present in individuals with high-risk endophenotypes who do not develop an ASD. Our aim, therefore, was to investigate the earliest age of identifiable differences in the social neural phenotype in infants at familial risk for ASD, and to do so using novel fNIRS methodologies. In order to examine endophenotypes associated with neural responses to both faces and emotional expressions, we placed fNIRS probes over right lateral and frontal brain regions, respectively, of infants who were shown movie clips of their mothers, and similar-looking strangers, changing from a neutral to smiling expression (Figure 1). This paradigm was used to examine the magnitude of responses (Analysis I) in 6- to 7-month-old infants at high-risk for developing autism (HRA), defined as having an older sibling with ASD, as well as matched low-risk controls (LRC).

As differences in signal at individual channels were expected to be subtle, we also sought new methods of analysis for detection of endophenotypes through response waveforms. We therefore employed independent component analysis (ICA) to examine the timing and distribution of the waveforms in our data that correspond to functional connectivity (Analysis II). Several approaches to the analysis of functional connectivity have been utilized with fNIRS and fMRI data, and more recent studies have examined the optimal methods for such analyses (Katura et al., 2008; Zhang et al., 2010). One practical problem arises from the ICA approach, however, and that is the assignment of separated components. In fMRI studies, independent components (ICs) can be correlated with known hemodynamic response functions, but these functions are only beginning to be described for fNIRS studies. Moreover, these responses may change over the course of infant development, and they cannot be assumed to be comparable when analyzing neurologically atypical populations. Several studies involving the use of ICA methods with fNIRS data have successfully identified components of interest without the use of hemodynamic models (Akgul et al., 2006; Katura et al., 2008; Zhang et al., 2010). We therefore adapted a method of extracting task-related ICs (Katura et al., 2008) in our second analysis in order to better describe neuronal activation signals and their distribution between face and emotion-processing regions in infants at high-risk for ASD as compared to controls.

## Results

### Analysis I: Face identity and emotion by group

**Figure 2:**
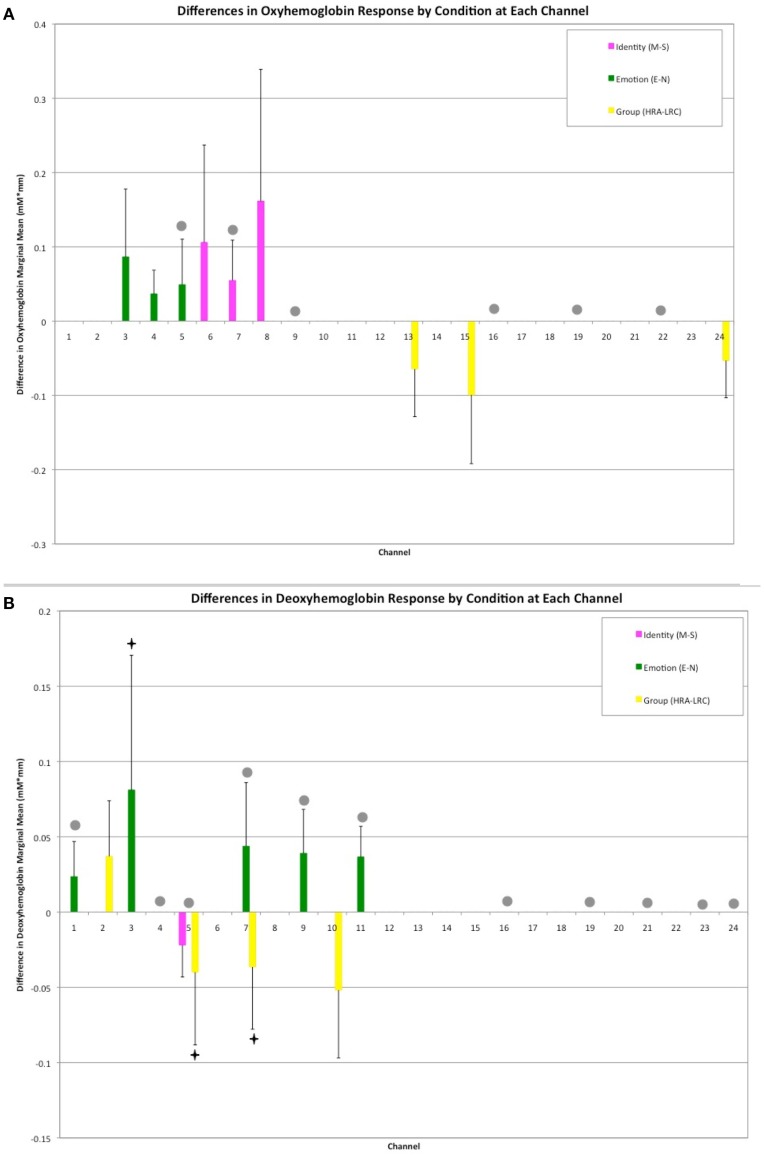
**Results of Face Identity × Emotion × Group ANOVA by Channel**. **(A)** Difference in Oxy-hemoglobin Response; **(B)** Difference in Deoxy-hemoglobin Response. (M = Mother, S, Stranger; E, Emotion; N, Neutral; HRA, High-Risk Autism; LRC, Low-risk Control). Channels with significant main effects of Face Identity (magenta) and Emotion (green) occurred in frontal regions, while the main effect of Group was seen in right lateral channels (yellow). The difference in population marginal means with corresponding 95% confidence intervals, Tukey’s HSD used for correction for multiple comparisons. Bonferroni correction was employed for correction of comparisons across channels. Marginally significant effects are indicated by “♦” (full statistics in Table A1 in Appendix). Significant interactions were seen across frontal and lateral channels (gray circles). Further information regarding interactions is available in Table 1.

**Figure 3:**
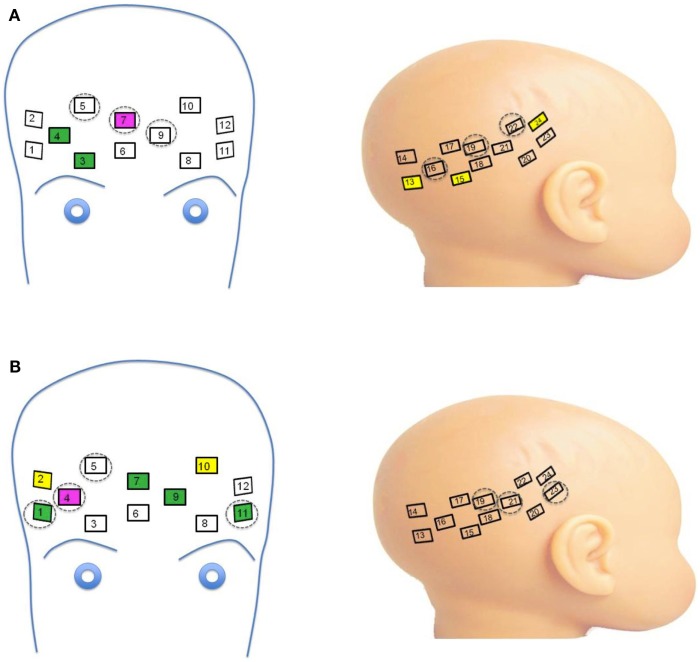
**(A)** Channels significant for main effects and interactions on oxy-hemoglobin response. Channels with main effects of Face Identity (magenta) and Emotion (green) occurred in frontal regions, while the main effect of Group was seen in right lateral channels (yellow). Significant interactions were seen across frontal and lateral channels (dashed circles). **(B)** Channels significant for main effects and interactions on deoxy-hemoglobin response. Channels with main effects of Face Identity (magenta), Emotion (green), and Group occurred in frontal regions, while significant interactions were seen across frontal and lateral channels neighboring those significant for oxy-hemoglobin responses (dashed circles).

A mixed effects ANOVA involving factors Face Identity (mother or stranger), Emotion (neutral or smiling), and Group (LRC or HRA) revealed significant main effects, as well as interactions at several channels (Figures 2A,B, see Table 1 and Table A1 in Appendix for full statistics). A main effect of emotion was seen in three right frontal channels, where oxy-hemoglobin responses to smiling were greater than those to neutral expressions (Figures 2A and 3A). Three left-sided frontal channels had deoxy-hemoglobin responses that were greater (more negative) to smiling faces than to neutral faces. A main effect of face identity was noted in centrally located frontal channels, where oxy-hemoglobin responses to mother were greater than to those for stranger (Figures 2A and 3A). One channel was significant for a greater deoxy-hemoglobin response to stranger than to mother. There were also seven channels demonstrating a main effect of group, with significantly different changes in oxy-hemoglobin by group in the postero-lateral right hemisphere, and significantly different changes in deoxy-hemoglobin by group in frontal regions. Oxy-hemoglobin responses in three right lateral channels were greater for the LRC group than the HRA group. The opposite pattern was seen in four frontal channels, where deoxy-hemoglobin responses were greater for the HRA group than the LRC group.

**Table 1:** **Results of face identity × emotion × group ANOVA by channel**.

Ch	Interactions
1	HbD: M > S for HRA group only
2	
3	
4	HbD: M > S for HRA group only
5	HbO: an interaction of Face Identity and Emotion in the LRC group showed greater HbO response to mother smiling than mother in the neutral condition as compared to the difference seen in the stranger condition. This effect was not significant in the HRA group
	HbD: S > M for HRA group only
6	
7	HbO: for LRC & E, M > S
	HbD: HRA > LRC for M response, S > M for LRC only
8	
9	HbO: M > S for LRC group only
10	
11	HbD: smiling was associated with a reduced HbD response for both mother and stranger in the HRA and LRC groups. There was an interaction effect of Face Identity and Emotion present only in the LRC group, and HbD responses to mother smiling were less in the LRC group than in the HRA group
12	
13	
14	
15	
16	HbO: M > S for LRC group only
	HbD: M > S for HRA group only
17	
18	
19	HbO: an interaction of Face Identity and Emotion in the LRC group showed greater HbO response to mother smiling than mother in the neutral condition. The opposite effect was true of the HRA group, in which HbO response to mother in the neutral condition was greater than to mother in the smiling condition
	HbD: S > M for LRC group only
20	
21	HbD: S > M for LRC group only
	HbD responses to mother in the neutral condition were greater for the HRA group than for the LRC group. An interaction effect in which the effect of the smiling condition reduced the HbD response to mother’s face was only seen in the HRA group
22	HbO: for LRC, M > S
23	HbD: M > S for HRA group only
	HbD responses to mother in the neutral condition were greater for the HRA group than for the LRC group. An interaction effect in which the effect of the smiling condition reduced the HbD response to mother’s face was only seen in the HRA group
24	HbD: S > M for LRC only

*M, Mother; S, Stranger, E, Emotion; N, Neutral; HRA, High-Risk Autism; LRC, Low-risk Control; HbO, oxy-hemoglobin; HbD, deoxy-hemoglobin; m, difference in population marginal means with corresponding 95% confidence intervals, Tukey’s HSD used for correction for multiple comparisons. Note that interactions are described by magnitude of the response regardless of direction. Bonferroni correction was employed for correction of comparisons across channels*.

Thirteen of the 24 channels were significant, or marginally significant, for interaction effects. These results are summarized in Table 1, and depicted in Figures 2 and 3. Oxy-hemoglobin responses across frontal and lateral regions revealed an interaction in which responses to mother were greater than those to stranger only for the LRC group; no effect of face identity was present for the HRA group. In addition, a significant three-way effect was found, in which the interaction of Face Identity and Emotion was significant for the LRC group, but not for the HRA group. An interaction of Group and Face Identity was seen for deoxy-hemoglobin responses in both frontal and lateral channels. For the HRA group, a greater deoxy-hemoglobin response was seen to mother as compared to stranger faces across many channels. Several lateral channels in the LRC group demonstrated a greater deoxy-hemoglobin response to the neutral mother as compared to other conditions, but this interaction effect was not seen in the HRA group. The region-specific effects and interactions are summarized in Figures 2B and 3B.

### Analysis II: ICA component clustering

**Figure 4:**
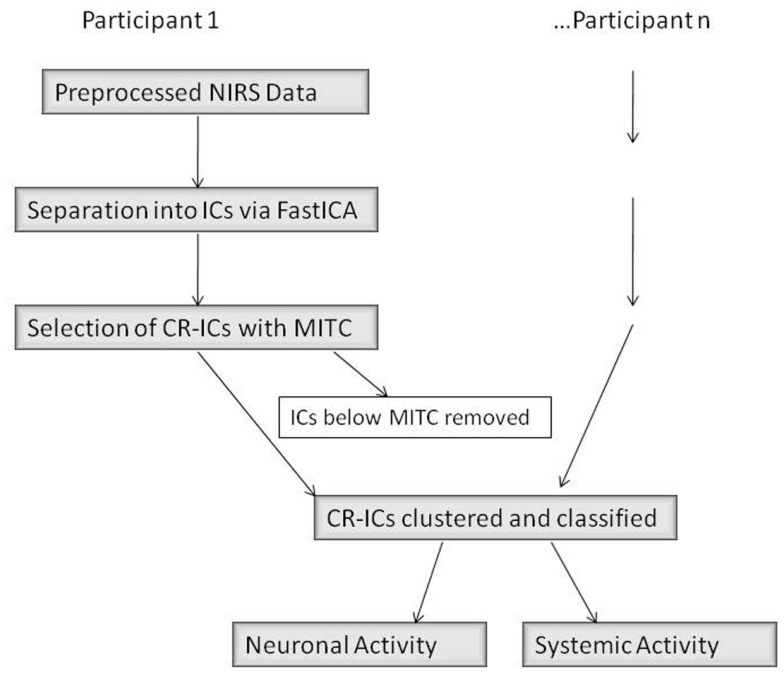
**ICA Data Analysis Diagram (adapted from Katura et al. (2008))**. IC, independent component; CR-IC, condition-related independent component; MITC, mean inter-trial cross-correlation.

There were three main steps in our connectivity analysis (Figure 4, adapted from Katura et al., 2008). In the first step, ICA was performed (FastICA v.2.5 – www.cis.hut.fi/projects/ica/fastica/) to separate components of fNIRS signals obtained from 24 channels. In the second step, ICs were selected based on mean inter-trial cross-correlation (MITC), which is based upon the idea that neuronal activation signals should be reproducible at each trial. A high MITC indicates a condition-related independent component (CR-IC). In the case of our experiment, “condition” referred to the emotional state of the face stimuli (neutral or smiling), as we were interested in analyzing the factor with potentially the greatest distribution of connected responses across lateral face processing and frontal emotion-responsive regions. As noted in the motor experiments conducted by Katura et al. (2008), systemic hemodynamic changes create global noise components while task-related neuronal activity should localize to specific regions of the brain. Systemic changes may, however, be associated with the task, and will therefore have a high MITC. Although the current experiment does not involve motor activity, such systemic changes may also occur due to activation of the autonomic nervous system. These changes are related to trials, but do not necessarily represent localized neuronal activation. A third step using *k*-means clustering methods was therefore employed to categorize CR-ICs into those representing neuronal activity involved in the two cognitive processes of face and emotion perception, as well as a third cluster of condition-related “noise.” Finally, we examined the response functions of these clusters, and classified them as frontal or lateral based upon a weight index.

### Analysis II: Centroid waveforms for oxygenated and deoxygenated hemoglobin

**Figure 5:**
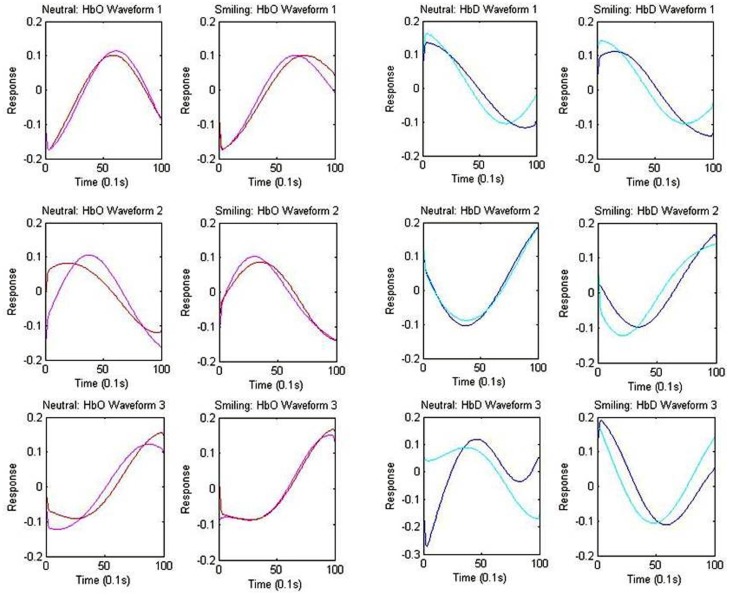
**Centroid Waveforms of Component Clusters**. Each waveform (centroid) is the mean of the components of the cluster, after centering and normalizing points to zero mean and unit standard deviation. Three waveforms were obtained for each of the oxy-hemoglobin (HbO: red = HRA, pink = LRC) and deoxy-hemoglobin (HbD: blue = HRA, cyan = LRC) components, for both the smiling and neutral conditions. Waveforms from the HRA and LRC groups are shown together based upon correlation.

The centroid waveforms associated with oxy-hemoglobin and deoxy-hemoglobin for each of the neutral and smiling conditions are depicted in Figure 5. Centroids for the HRA and LRC groups were plotted together for comparison based upon correlation of waveform. The three centroids, generated separately for each group, were very similar in timecourse. As the centroids represent a normalized response, however, this does not yield information about the differences in magnitude of the response. In the case of oxy-hemoglobin, Waveform 1 (Figure 5) follows a timecourse with a peak at 6–8 s following stimulus presentation. This waveform remained relatively unchanged during the neutral and smiling conditions. A second oxy-hemoglobin waveform had similar properties, but peaked much earlier, in the 3–4 s range. Finally, a third waveform appeared to peak very late, or even after the 10-s time window.

The results of deoxy-hemoglobin component clustering paralleled those of oxy-hemoglobin, in that the shape of waveforms was notably similar between HRA and LRC groups. In addition, for the LRC group, the timing of the first two deoxy-hemoglobin waveforms (Figure 5) mirrored the timing of oxy-hemoglobin waveforms, with the minimum value for deoxy-hemoglobin Waveform 1 and Waveform 2 occurring just after the maximum value for the respective oxy-hemoglobin waveforms. Notably, there was a time delay of approximately 1.9 s between deoxy-hemoglobin waveforms for the LRC and HRA groups that was significant for Waveforms 1 and 2 in the smiling condition [Waveform 1: *p* = 0.021, 95% CI (0.31 s 3.69 s); Waveform 2: *p* = 0.026, 95% CI (0.24 s 3.61 s)]. The deoxy-hemoglobin Waveform 3 for the neutral condition contained very few components, and is likely due to systemic noise.

### Analysis II: Examination of components within clusters

**Figure 6:**
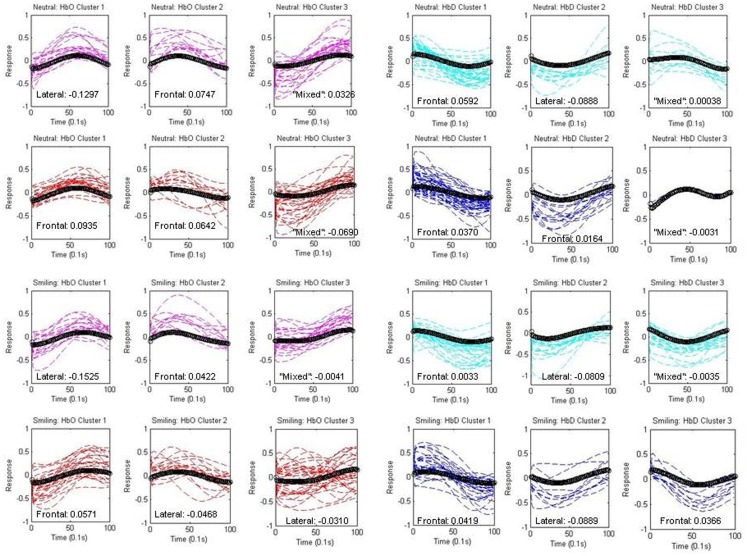
**Components within Clusters**. Individual components assigned to each centroid (black) are depicted separately for each group (HRA and LRC) and condition (neutral and smiling). Oxy-hemoglobin (HbO) components are shown in red (HRA) and pink (LRC), while deoxy-hemoglobin components (HbD) are shown in blue (HRA) and cyan (LRC). The spatial assignment by laterality index is indicated as “frontal,” “lateral,” or “mixed” for each cluster, and numeric differences in mean normalized weight across regions are given (Frontal-Lateral).

The individual components assigned to each cluster are depicted separately for each group and condition in Figure 6, along with the centroid waveforms. A laterality index (LI) was employed using the normalized spatial weights of each component within the cluster in order to assign the waveform to a frontal or lateral region.

$$LI=\frac{\sum_{F}\hat{A}-\sum_{L}\hat{A}}{\sum_{F}\hat{A}+\sum_{L}\hat{A}}$$

This assignment is described on each graph in Figure 6, along with the difference in mean normalized weight across the channels in each region (Frontal-Lateral).

Oxy-hemoglobin Cluster 1 (corresponding to Waveform 1) was assigned to right lateral regions, and oxy-hemoglobin Cluster 2 (corresponding to Waveform 2) was assigned to frontal regions for the LRC group. The difference between mean weight increased for Cluster 1, and decreased for Cluster 2, in response to smiling. The distance from components to the centroid was significantly greater for the neutral condition as compared to the smiling condition for LRC Cluster 2 [*p* = 0.0079, 95% CI (0.0829, 0.5388)]. The third oxy-hemoglobin cluster was labeled as “mixed,” for having a very low LI and difference of mean weight between regions. A different pattern emerged for the HRA group, with oxy-hemoglobin Cluster 1 assigned to frontal regions, and a reduction in LI with smiling. HRA Cluster 2 changed regional assignment from frontal to lateral between neutral and smiling conditions, respectively, and no significant differences in the distance between components and centroid were present.

Deoxy-hemoglobin Cluster 1 was assigned to frontal regions for both LRC and HRA groups. Cluster 2 maintained a constant right lateral index across conditions within the LRC group, but for the HRA group, this cluster changed from a slightly frontal index in the neutral condition to a right lateral index in the smiling condition. This is similar to the pattern of oxy-hemoglobin Cluster 2 for the HRA group. Cluster 3 in the neutral condition contains very few components, and due to the unusual shape and non-specific location of both HRA and LRC waveforms, is likely non-neural in origin. In the smiling condition, deoxy-hemoglobin Cluster 3 demonstrates a decrease that may represent a widespread pattern of deoxy-hemoglobin change across cortical regions. This Cluster is assigned to frontal regions only for the HRA group.

Deoxy-hemoglobin Cluster 1 is also notable for the variability in the rise and fall of deoxy-hemoglobin component time series as compared to the centroid for the HRA group. An analysis of variance in the distance between components and the centroid for this cluster revealed that there is significantly greater variance for the HRA group as compared to the LRC group [*F*(1, 64) = 1.73, *p* = 0.03, 95% CI (1.06, 2.84)]. In addition, an increase in frontal weight and channel distribution occurred only for the HRA group during the smiling condition, causing a greater mean weight across frontal channels as compared to neutral conditions [*p* = 0.0002, 95% CI (0.01437, 0.4609)].

## Discussion

To our knowledge this study is the first to use fNIRS to distinguish infants at high-risk for autism from LRC. The fNIRS method involves a measure of metabolic activity associated with neural responses, linking infant studies to fMRI findings in older individuals, thus the significance of this investigation is that it can provide a key addition to our understanding of not only the timing of developmental processes involved in autism risk, but also the broader range of face processing endophenotypes that may be present during infancy.

The OFC is implicated in attention to social stimuli, the perception of facial emotion (Leppanen et al., 2007) and in the regulation of autonomic responses to social situations (Schultz et al., 2000; Dalton et al., 2005; Goodkind et al., 2011). Studies involving fNIRS have revealed the activation of the frontal and prefrontal cortex in the processing of social cognition and facial emotions (Blasi et al., 2011; Minagawa-Kawai et al., 2009; Grossmann et al., 2010). In an ERP study of infant social development by Johnson (2005), suprasylvian structures and prefrontal cortex were found to play a key role in face responses, and the early social brain network. Further, EEG data have demonstrated that high-frequency oscillations in the gamma-band (20–100 Hz) are elicited in the right prefrontal cortex to a greater extent in response to direct gaze from a face than in response to averted gaze (Farroni et al., 2007; Grossmann et al., 2007). Likewise, Minagawa-Kawai et al. (2009) characterize the observed fNIRS response to mothers in OFC as the neural correlates of “enhanced emotional valence” in cues given by a known social agent. Our results are consistent with prior studies, in that oxy-hemoglobin responses to the effect of smiling were noted in right frontal cortex in a region most likely representing OFC. It is therefore likely that the frontal responses we have measured through both oxy- and deoxy-hemoglobin changes create a broader picture of an attentive response that is particularly tuned to social positive affect.

The main effect of group revealed significantly greater oxy-hemoglobin responses in lateral regions for LRC as compared to HRA, and significantly greater deoxy-hemoglobin responses in frontal channels for HRA as compared to LRC. There were also numerous interaction effects between Group and Face Identity, generally revealing a greater deoxy-hemoglobin response to mother’s face as opposed to stranger face within the HRA group across both frontal and lateral regions. The interactive effect of Face Identity on the greater oxy-hemoglobin response to smiling versus neutral conditions was present only for the LRC group, indicating that while the HRA group demonstrated an oxy-hemoglobin response to smiling in OFC, the difference in response to the smile of mother and the smile of stranger faces was not significant. Returning to the role of OFC within the complex social brain network, this lack of interaction between identity and emotional state in the HRA group might indicate a lack of selective attention to a known social agent. At the same time, the deoxy-hemoglobin changes seen only in the HRA group indicate that some level of discrimination between mother and stranger is occurring.

Despite group differences in the magnitude of the oxy-hemoglobin response to face identity, the waveforms identified for both HRA infants and controls were remarkably similar. The waveform most consistent with the timecourse of face responses was weighted toward a frontal distribution in the HRA group, and a right lateral distribution in controls. This may be due to a difference in the hemodynamic response function of frontal regions in infants at high-risk for autism, where patterns typically seen only in right lateral regions in controls are dominant in frontal areas of the at risk brain.

One prominent feature of HRA hemodynamics, however, was the deoxy-hemoglobin differential response to mother as compared to stranger. Taga et al. (2003) observed that while deoxy-hemoglobin responses were not significant on a group-level in response to visual stimuli, individual subjects demonstrated a significant decrease in deoxy-hemoglobin that accompanied oxy-hemoglobin responses. The timing of this response was similar to that of oxy-hemoglobin, but reached a minimum value just after the peak of the oxy-hemoglobin timecourse. In Analysis I, deoxy-hemoglobin responses were reduced for stranger as compared to mother in right lateral regions of the LRC group, while the opposite effect was significant in the HRA group. Two channels in the suprasylvian region were significant for a deoxy-hemoglobin response to mother in the neutral condition that was greater for the HRA group than for the LRC group, as well as an effect of the smiling condition that reduced this response to mother’s face. The fMRI responses of right lateralized structures associated with face recognition have been shown to be hypoactive in autistic individuals (McPartland et al., 2004; Dalton et al., 2005; Hadjikhani et al., 2007; Corbett et al., 2009; Dziobek et al., 2010; Monk et al., 2010), with hyperactivity in orbitofrontal regions and amygdala in response to specific facial features (Dalton et al., 2005). Our results are consistent with the work by Dalton et al. (2005), which identified a region in the right fusiform to occipital cortex associated with greater activation for familiar faces than for unfamiliar faces only for a control group when compared to adolescents with ASD. The autistic group showed greater activation in the left OFC specifically in response to the emotional content of the faces and not to faces in general, with corresponding right amygdala activation that was not specific to facial emotions, but a response to faces in general (Dalton et al., 2005). Since deoxy-hemoglobin has been implicated in the early hemodynamic responses as measured by BOLD fMRI, the regions of signal described by Dalton et al. (2005) may indeed correspond to those localized in the present study. This study takes this work further by demonstrating that a similar atypical response pattern is seen in siblings at risk for ASD and is present very early in development, long before the onset of behaviors associated with autistic symptoms.

Our second analysis further supports the hypothesis that measures of deoxy-hemoglobin reveal distinct patterns between groups. From Analysis II, we can conclude that both HRA and LRC groups demonstrate at least two hemodynamic waveforms associated with the processing of faces and emotions, and that these waveforms can be localized to frontal and lateral regions. These waveforms are similar in shape in both groups, however subtle differences in timing and distribution may in fact relate to an underlying endophenotype. The difference in the timing of deoxy-hemoglobin waveforms in the smiling condition between LRC and HRA groups is consistent with the findings of a difference in deoxy-hemoglobin response corresponding to the mother smiling condition. In addition, the initial increases in deoxy-hemoglobin seen in Waveform 1, as well as its heavily frontal distribution in the HRA group, imply that this element of the hemodynamic response differs between the HRA and LRC groups within frontal regions. Taken together, these findings may indicate a difference in frontal lobe hemodynamic responses between the LRC and HRA groups, as well as a difference in how these frontal responses connect to other regions of the brain. In addition, the differences in spatial weight of the waveforms associated with face processing may indicate greater recruitment of frontal regions during the perception of faces in infants at high-risk for autism.

It is important to note that only 20% of infants defined as “high-risk” will go on to develop ASD, so while our observations indicate differences from LRC, they cannot necessarily be interpreted as markers of difference in cognitive perception. Anatomical studies frequently implicate the frontal lobes in the pathophysiology of autism (Courchesne et al., 2001, 2011; Schmitz et al., 2007; Amaral et al., 2008), and such frontal lobe variation may exist in unaffected siblings, as well (Barnea-Goraly et al., 2010). As these differences in morphometry are most notable in measures taken from early childhood, it is likely that the greatest period for detection of change occurs within the first 6–14 months (Courchesne et al., 2005, 2011), coinciding with a period of significant cortical remodeling. Our results show significant differences in the timing of deoxy-hemoglobin waveforms between LRC and HRA groups at 6 months of age, as well as differences between the waveforms of oxy-hemoglobin responses localized to frontal regions. In anatomical studies, the frontal lobes have shown the greatest degree of enlargement in individuals with autism (Carper et al., 2002), as well as increased neuronal cell counts (Courchesne et al., 2011). It is possible that such structural differences in this region could affect the hemodynamic response profile in infants at risk for developing ASD, without necessarily causing the ASD phenotype.

The differences we have observed between the high versus low-risk infants in hemodynamic activity may reflect a response profile of infants at risk for ASD, though they may not necessarily cause the ASD phenotype. (As our sample grows in size and crosses the threshold for a definitive diagnosis of autism – 36 months – we will eventually be able to ascertain whether our fNIRS measures *predict* autism as well as serve as an endophenotype for the disorder.) Our results may therefore represent a complex relationship between structural differences and cognitive function within the HRA group, characterized by changes in frontal lobe hemodynamics, which may in turn be correlated with reduced connectivity between regions. These differences in neuronal architecture may also be associated with states of hyperarousal, however further studies are required to determine a causal relationship between structure, function, and behavior.

Of note, we expect only one in five high-risk infants to eventually meet criteria for an ASD (Ozonoff et al., 2011). The fact that we have observed such robust group differences at 6 months of age suggests that regardless of eventual outcome, the majority of high-risk infants carry an endophenotype of ASD that involves subtle differences in frontal and temporal hemodynamic response to faces It is therefore important to follow the entire cohort of infants into childhood, in order to examine factors that may lead to the disorder, as well as attributes which provide compensation for an early endophenotype.

## Materials and Methods

### Participants

Twenty-five 5- to 7-month-old infants (14 female, mean age = 7 months, age range: 6.33–7.97 months) participated in the study. As noted by Ozonoff et al. (2011), one in five infants having an older sibling diagnosed with ASD will go on to develop the disorder. Ten of these infants (6 female, mean age = 7.06 months, age range: 6.33–7.97 months) had an older sibling with a diagnosis of ASD, and were thus defined as being at high-risk for ASD (HRA group). The other fifteen infants were recruited as LRC (group) for comparison, and were defined as such by having at least one older sibling and no immediate relatives with a diagnosis of ASD. The probands of the HRA infants all received community diagnoses of ASD, which were confirmed using the Social Communication Questionnaire (SCQ) and Autism Diagnostic Observation Schedule (ADOS) administered by trained staff.

Of the low-risk control infants recruited, three were excluded from the analysis due to insufficient data (two could not complete the required number of trials due to fussing, and one had data with significant artifacts). Two were additional control subjects that could not be matched to infants in the HRA group. Ten of the control infants (6 female, mean age = 6.91 months, age range: 6.4–7.93 months) were selected for analysis based upon gender and behavior during the task (Table 2). All infants included in the experiment were: (1) born after 36 weeks gestational age, (2) born weighing more than 2500 g, and (3) born without a known neurological or uncorrected visual abnormality.

**Table 2:** **High-risk autism (HRA) group and low-risk controls (LRC)**.

	Age (months)	Gender	AVERAGE number of trials	Average number of neutral/smiling	Average number of mother/stranger
High-risk autism	7.06	6 Female; 4 male	13.4	12.9/11.9	6.1/6.4
Low-risk control	6.91	6 Female; 4 male	13.4	13.2/12.8	6.4/6.6

Experiments were conducted under approval by the Institutional Review Board at Children’s Hospital Boston, Boston University, and Massachusetts Institute of Technology, and in accordance with the Declaration of Helsinki. Written informed consent was obtained from the parents of all infant participants.

### Stimuli

Prior to fNIRS imaging, we created a high-definition digital video recording of the mothers of participants as a movie stimulus. Mothers were asked to stand against a white background, and were draped with a white cloth. They were instructed to answer a series of questions while their faces were being recorded on video, with the purpose of creating a natural movement of the mouth. Mothers performed this task twice: first with a neutral facial expression, and a second time with a smiling expression (Figure 1A). Sound was removed from the videos in order to eliminate any multisensory effects of voice. Videos were then edited to obtain 16 s clips under each of the neutral and smiling conditions, and to combine these clips into a continuous 32 s video trial with transition from neutral to smiling expression.

### fNIRS task procedures and equipment

Infants were seated on a parent’s lap throughout the experiment, and passively observed the stimuli. Visual stimuli were presented on a 17-inch Tobii T-120 eyetracker monitor at a distance of approximately 60 cm from the infant. A visual baseline of moving objects was used for at least 10 s at the beginning of each session to achieve a baseline measurement and to encourage visual attention to the screen. We monitored and recorded eye gaze throughout the experiment, and presented stimuli only while infants were attending to the display. Infants were presented with seven trials each of the video of their mothers and the video of a stranger. The videos of other participants’ mothers and women unknown to the infant were used for the stranger condition, and were selected for each infant based upon similarity to the mother, including hair and eye color, ethnicity, position of facial features, and movement of the mouth during speaking and smiling. Infants were randomized to one of two semi-random orders of video presentation (mother or stranger video), for a total of 14 trials presented to each infant, counterbalanced across participants. A fixation cross was present between trials (Figure 1A) for 5 s or until infants attended to the center of the screen. Conditions (neutral or smiling) within each trial were excluded if infants viewed fewer than 1.5 s of the first 5 s of the condition. This ensured that a 10 s time window could be obtained in which the trial stimulus was present on the screen.

The Hitachi ETG-4000 fNIRS system with 24 simultaneously recording channels (as described in Supplemental Methods Information) was used to collect hemodynamic response during stimulus presentation. A cap was designed for infants in order to affix the fNIRS optical probes to the frontal and right lateral portions of the head (Figure 1B). The design consisted of three neoprene panels, each of which could hold one of the two sets of probes, and which could be adjusted with velcro closures to allow for individualized placement of the probes once on the infant’s head. The neoprene fabric created support for placing the probes over frontal cortex, while maintaining the level of elasticity necessary for conforming to the infant head, and thus reduced noise due to artifact. The frontal panel was centered in the nasion–inion line, with the inferior frontal probes positioned directly above the eyebrows, in a direction parallel to the T3-Fp1-Fp2-T4 line in the international 10–20 system as described by Minagawa-Kawai et al. (2009). The right lateral panel was positioned so that the anterior portion of the panel was located just superior to the right ear, with the panel extending toward the occiput (Figure 1B).

Based on the light intensity detected through each channel, relative concentrations of oxygenated and deoxygenated hemoglobin were calculated from absorbance at each wavelength using the modified Beer–Lambert law. This conversion, as well as further data analyses, were implemented through a customized Matlab script (version 7.6, Mathworks Inc., Natick, MA, USA).

### fNIRS data pre-processing

Time series corresponding to oxy- and deoxy-hemoglobin values were first processed using a third order Butterworth filter between 0.01 and 0.1 Hz, and additional artifacts were identified and extracted if the raw signal exceeded a value of 4.95, or if total hemoglobin change exceeded 0.1 mM × mm within a 0.2 s time window. The first 10 s of resting data (recorded prior to the experimental task) were removed to eliminate artifacts from initial stabilization of the signal, and linear trends were then removed from the entire time series.

### Analysis I

For each participant, each trial condition was included if 1.5 s of video was viewed within the first 5 s of the condition, and no significant motion occurred. This allowed for use of a 10 s observation window within each condition, which would include the peak of the response as described in the literature (Taga et al., 2003; Minagawa-Kawai et al., 2009). In their measures of orbitofrontal infant responses to mother and stranger videos, Minagawa-Kawai et al. (2009) found significant group differences between mother and stranger trials with inclusion of 2–3 trials of each, however given our need to compare between groups, in our analyses we included only infants who viewed at least five of the seven videos of each trial and condition pairing with no significant artifacts after initial filtering. Linear trends were then removed from the data. For each subject, the data were parsed into 10 s time windows with 0.1 s time resolution beginning at the first instance of 1.5 s of looking within the first 5 s of each condition presentation. These time windows were corrected to a baseline value at the onset of each time window in order to create a zero baseline for comparison of conditions. As our stimuli included videos in which the face changed from a neutral to a smiling expression, it is possible that an unequal visual baseline period could precede these two emotion conditions. While this limited our ability to independently analyze responses to each of the emotion conditions, our study aimed to examine only those effects unique to each emotion, and how those effects differed between groups. Following this correction, trials were grouped by emotion condition (neutral or smiling) as well as by identity (mother or stranger) and averaged to obtain a mean value for oxy- and deoxy-hemoglobin change at individual channels. Mean values at each time point from each subject were also pooled to produce a group mean value by condition at each channel and time point. Channels from individual subjects with low oxy-hemoglobin signal-to-noise (Mean/Standard Deviation < 1.0) were excluded from the group average. An oxy- or deoxy-hemoglobin response was defined as the difference between the maximum value in the latter 5 s of each 10 s time window and the minimum value in the first 2 s of a window. Participants viewed an average of 7.1 s of the 10 s windows, with no significant difference in viewing time between condition or group.

### Analysis II

The acquired functional fNIRS data had a relatively large number of temporal samplings as compared to spatial samplings of signal (10 Hz sampling rate at each of 24 channels), therefore temporal ICA was employed. This method assumes a temporal independence of sources, which can only be a partially valid assumption when considering the connectivity of the brain and condition-dependent responses. Nevertheless, this method has demonstrated convincing results in many task-related fNIRS studies, with care taken in the interpretation of independence among sources (Morren et al., 2004; Katura et al., 2008; Markham et al., 2009). Further explanations of temporal ICA involving fNIRS measurements can be found in Section “Supplemental Methods Information.”

In this analysis, we utilized FastICA v2.5 (www.cis.hut.fi/projects/ica/fastica/) to conduct the ICA decomposition algorithm (Hyvarinen, 1999). ICA analysis was separately performed on the pre-processed individual oxy-hemoglobin and deoxy-hemoglobin data sets. Parameter settings included: approach = “deflation,” non-linearity = “skew,” stabilization = “on,” fine-tune = “on,” maximum number of iterations = 10000, epsilon = 0.00001, initial value = “random.” These were based upon previous use of FastICA with fNIRS data (Zhang et al., 2010). Prior to running the algorithm, principal component analysis (PCA) reduction was performed on the data from 24 channels to reduce the data dimensionality for each subject. The number of retained principal components was determined according to the minimum number of principal components that retained more than 99% of data variance (van de Ven et al., 2004; Zhang et al., 2010). The reduced data for each individual was put into ICA decomposition with the number of ICs equal to the number of the retained principal components. This yielded an average of 14.7 (SD = 2.4) oxy-hemoglobin components and 13.9 (SD = 1.9) deoxy-hemoglobin components in the HRA group; 14 (SD = 2.4) oxy-hemoglobin components, and 14 (SD = 2.4) deoxy-hemoglobin components in the LRC group.

### Condition-related component selection

For each participant, each trial condition was included if 1.5 s of video was viewed within the first 5 s of the condition, and no significant motion occurred in the original data. This allowed for use of a 10 s observation window within each condition, which would include the peak of the response as demonstrated by Analysis I in both orbitofrontal and lateral face processing regions, and which would be suitable for MITC.

For each subject, the ICs were parsed into 10 s time windows with 0.1 s time resolution beginning at the first instance of 1.5 s of looking within the first 5 s of each condition presentation. These time windows were corrected to a baseline value at the onset of each stimulus. Following this correction, trials were grouped by emotion condition (neutral or smiling) at each channel. Smoothing with a Gaussian kernel (FWHM 2s) was applied to each trial in order to calculate MITC.

The selection criterion for CR-ICs was data-based rather than based upon hemodynamic models, and included those components with a MITC greater than the mean value of MITCs within each subject. This allowed for individual differences in inter-trial reproducibility of waveforms, while eliminating components that were likely the result of artifact (Figure 4).

### Clustering of condition-related components

In the third step of our analysis, we used *k*-means clustering methods to categorize CR-ICs into three groups based upon waveform. As activation specific to faces was hypothesized to localize to right lateral cortex, and activation associated with the emotion condition appeared to cluster in frontal regions, it was hypothesized that two different hemodynamic response functions could underlie these cognitive processes. This hypothesis was based upon the finding that fNIRS hemodynamic responses can differ between brain regions, even in the adult (Jasdzewski et al., 2003). A third cluster was included to account for any task-related systemic activity (Franceschini et al., 2000; Boas et al., 2002; Katura et al., 2008). As the sign of individual ICs is randomly determined, CR-ICs were each normalized and assigned a sign so that correlation with the boxcar waveform of trials became positive. The distance minimized in the clustering process was one minus Pearson’s correlation coefficient. Each cluster centroid was therefore the mean waveform of the components of the cluster, after centering and normalizing those points to zero mean and unit standard deviation.

The estimated spatial weights (see Supplemental Methods Information) of each component within each subject were then normalized for cluster analysis. The normalized weights of each component at each waveform cluster were averaged in order to examine the distribution of the waveform. This method of analyzing spatial distribution has been validated in prior work, with comparison to both group-level *t*-maps and seed correlation (Zhang et al., 2010).

### Supplemental methods information

#### Near-Infrared Spectroscopy

Near-Infrared Spectroscopy is a FDA approved technology that has been used for the past 20 years with adults and for the past 10 years with infants (Taga et al., 2003; Aslin and Mehler, 2005; Lloyd-Fox et al., 2010). The fNIRS imaging technique is able to measure oxygen levels based on the principle that hemoglobin absorbs near-infrared wavelengths of light in proportion to the concentration levels of oxygen in the blood. We utilized the Hitachi ETG-4000 fNIRS system with two wavelengths of light (695–830 nm) to measure cortical levels of oxy-, and deoxy-hemoglobin. The near-infrared light was guided by optical fiber bundles that were 1 mm in diameter. On the ETG-4000 device, each pair of adjacent incident and detection fibers defined a single measurement channel. The fNIRS probes consist of two 3 × 3 chevron arrays, each with five emitting and four detecting fibers held in place by a silicone support with 3 cm spacing.

#### Temporal independent component analysis of fNIRS data

Temporal ICA is classically explained in terms of the “cocktail party” problem (Bassett et al., 1953; Hyvarinen et al., 2001). In a crowded cocktail party, many people are talking at the same time. If several microphones are present, then their outputs are different mixtures of voices. Given such mixtures, ICA identifies the original voices from the mixtures by assuming that the original voice signals are independent from each other.

In the case of fNIRS measurements, measurements of *T* time points from *N* channels *x*(*t*) = [*x*_1_ (*t*), *x*_2_ (*t*),  …, *x_N_* (*t*)]^*T*^ : (*t* = 1, 2,  …, *T*) are comparable to the mixtures recorded by microphones, and the *N* “true” sources *S*(*t*) = [*S*_1_ (*t*), *S*_2_ (*t*),  …, *S_N_* (*t*)]^T^ : (*t* = 1, 2,  …, *T*) can be viewed as the understandable voice signals from each individual. The fNIRS measurements at each channel can thus be expressed as a mixing procedure by multiplying matrix **A**:

X(t)=As(t)

Independent component analysis decompositions can be viewed as an inversion of this:

$$Ŝ\left(t\right)=W\text{x}\left(t\right),$$

where **ŝ**(*t*) is an estimation of the true sources, and **W** is an unmixing matrix. The pseudoinverse of **W** is an estimation of **A**:

$$\hat{A}=W^{-}.$$

**Â** therefore represents a set of the spatial weights, which can be used to localize sources to brain regions (Zhang et al., 2010), and **ŝ**(*t*) represents a set of the temporal activities related to those regions.

## Conflict of Interest Statement

The authors declare that the research was conducted in the absence of any commercial or financial relationships that could be construed as a potential conflict of interest.

## Figures and Tables

**Figure A1:**
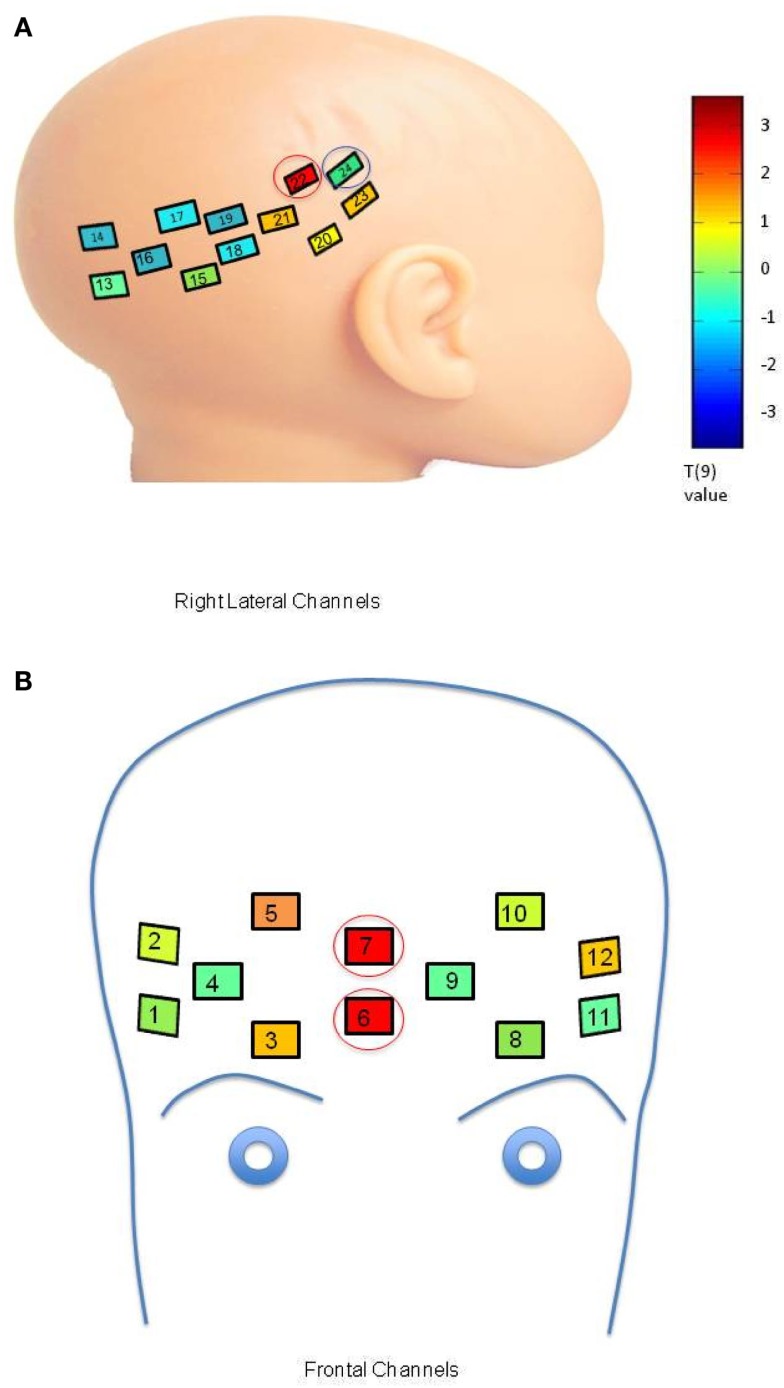
**(A)** Comparison of response to neutral mother and neutral stranger faces within the LRC group. Channel placement and numbers are shown on the right lateral portions of the infant head. Colors within the channels indicate a *T*-value map across 10 LRC subjects comparing oxy-hemoglobin response to the neutral mother’s face as compared to the neutral stranger’s face at each channel location (color bar, *p* = 0.05, Bonferroni corrected). The channel circled in red indicates significant differences in oxy-hemoglobin response, and the channel circled in blue indicates significant differences in deoxy-hemoglobin response. **(B)** Comparison of Oxy-hemoglobin Response to Smiling Mother and Smiling Stranger Faces within the LRC group. Significant activation occurred in channels placed on the front of the head (numbered as shown). Colors within the channels indicate a *T*-value map across 10 LRC subjects comparing oxy-hemoglobin response to the smiling mother’s face as compared to the smiling stranger’s face. Channels circled in red indicate significant differences in oxy-hemoglobin response.

**Figure A2:**
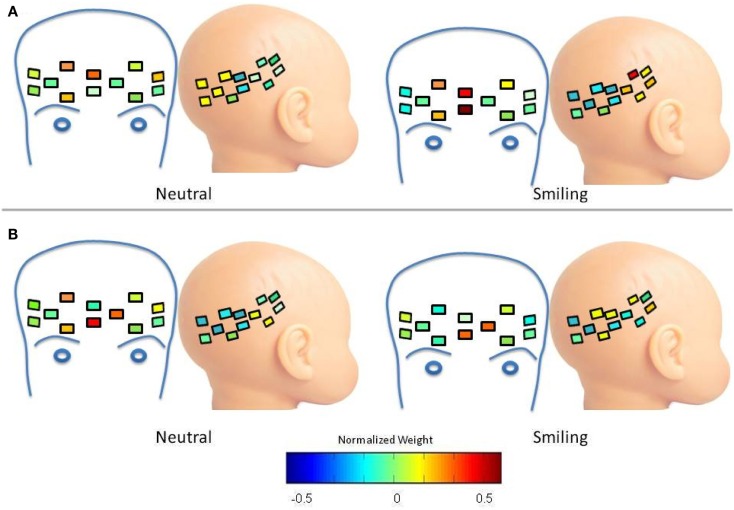
**Spatial distribution of oxy-hemoglobin waveform 2**. Colors within channels indicate normalized weights for this waveform according to the color bar. **(A)** LRC Group. Compared to the neutral condition, there is an increase in right lateral distribution of Cluster 2 during the smiling condition, and a focally increased frontal weight. **(B)** HRA group. There is increased right lateral distribution of Cluster 2, but without a focal increase of weight. The spatial distribution of frontal weights is reduced between the neutral and smiling conditions.

**Figure A3:**
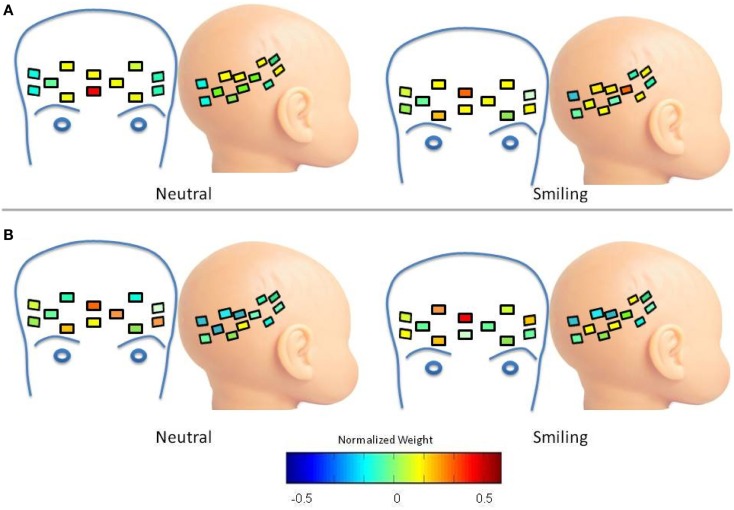
**Spatial distribution of deoxy-hemoglobin waveform 1**. Colors within channels indicate normalized weights for this waveform according to the color bar. **(A)** LRC Group. The waveform has greater frontal weight in the neutral condition, and is distributed between focal frontal and right lateral regions in the smiling condition **(B)** HRA Group. Frontal weight increases during the smiling condition, resulting in a greater mean weight across frontal channels for the smiling as compared to neutral conditions (*p* = 0.0002).

## Appendix

### Analysis of mother versus stranger face responsiveness

An initial set of analyses aimed to identify channel-specific differences between mother and stranger conditions in the LRC group. Trials of the neutral and smiling conditions were initially separated for analysis in order to examine the difference between mother and stranger conditions without the effect of emotion. The responses to neutral mother and stranger faces were averaged over each trial and subject, and compared using a paired *t*-test (Figure A1A, *T*-value map, Bonferroni corrected). In the low-risk control group, oxy-hemoglobin responses to neutral mothers was significantly greater than to neutral strangers in one lateral channel (Figure A1A, channel 22, mean difference = 0.1472 mM × mm, 95% CI (0.008, 0.1552), *p* = 0.0482, Bonferroni). This lateral channel corresponded to the postero-lateral region of the right hemisphere identified as responsive to faces in Chapter 3 (Figure A1A, channel circled in red). A significant difference in deoxy-hemoglobin was also seen in a neighboring lateral channel, in which the decrease in response to stranger was greater than the decrease in response to mother [Channel 24: mean difference = 0.0888 mM × mm, 95% CI (0.0419, 0.1357), *t*(9) = 4.37, *p* = 0.0024, Bonferroni; Figure A1A, channel circled in blue].

In the smiling condition, the difference in oxy-hemoglobin response between mother and stranger trials was significant in two right-sided central frontal channels for the LRC group [Figure A1B, channel 6: mean difference = 0.1022 mM × mm, 95% CI (0.0702, 0.1342), *p* = 0.0036, Bonferroni; channel 7: mean difference = 0.1414 mM × mm, 95% CI (0.0006, 0.2821), *p* = 0.0492, Bonferroni; channels circled in red]. These frontal channels correspond to regions identified by Minagawa-Kawai et al. (2009) and Grossmann et al. (2010) as responsive to social and emotional stimuli.

### Spatial distribution of specific oxy-hemoglobin and deoxy-hemoglobin waveforms

As mentioned previously, oxy-hemoglobin Cluster 2 (corresponding to Waveform 2) was assigned to frontal regions for the LRC group, and the difference between mean weight of this cluster decreased in response to smiling. The spatial distribution of this change is depicted in Figure A2A. The decrease in difference between the mean weight of frontal and lateral regions can be interpreted as an increase in the right lateral weights of Cluster 2 during the smiling condition, and a focally increased frontal weight. There is also increased right lateral distribution of Cluster 2 for the HRA group Figure A2B, with a reduction in the spatial distribution of frontal weights between the neutral and smiling conditions.

In the case of LRC deoxy-hemoglobin Cluster 1, the waveform moves from a greater frontal weight in the neutral condition, to a distribution between focal frontal and right lateral regions in the smiling condition (Figure A3A), possibly indicating a connected pattern of response in these two regions. This is not the case for the HRA group, where an increase in frontal weight and channel distribution occurs during the smiling condition, causing a greater mean weight across frontal channels for the smiling as compared to neutral conditions (*p* = 0.0002, 95% CI (0.01437, 0.4609); Figure A3B).

**Table A1:** **Statistical results of face identity × emotion × group ANOVA**.

Ch	Identity	Emotion	Group (HRA or LRC)	Interactions
1		HbD: *F*(1, 72) = 4.08, *p* = 0.047		HbD: group × identity [*F*(1, 72) = 10.48, *p* = 0.0018]
2			HbD: *F*(1, 69) = 3.97, *p* = 0.05	
3		HbO: *F*(1, 66) = 3.66, *p* = 0.05		
4	HbD: *F*(1, 68) = 4.28, *p* = 0.042	HbO: *F*(1, 68) = 5.28, *p* = 0.0247		HbD: group × identity [*F*(1, 68) = 5.88, *p* = 0.018]
5		HbO: *F*(1, 62) = 2.59, *p* = 0.1		HbO: identity × emotion × group [*F*(1, 62) = 6.96, *p* = 0.0105]
				HbD: group × identity [*F*(1, 62) = 4.15, *p* = 0.046]
6	HbO: *F*(1, 56) = 2.65, *p* = 0.1			
7	HbO: *F*(1, 68) = 4.04, *p* = 0.048	HbD: *F*(1, 68) = 4.25, *p* = 0.04	HbD: *F*(1, 68) = 2.95, *p* = 0.09	HbO: identity × emotion × group [*F*(1, 68) = 4.37, *p* = 0.04]
				
9		HbD: *F*(1, 69) = 7.2, *p* = 0.009		HbO: group × emotion [*F*(1, 69) = 5.46, *p* = 0.022]
10			HbD: *F*(1, 63) = 5.43, *p* = 0.02	
11		HbD: *F*(1, 71) = 12.97, *p* = 0.0006		HbD: identity × emotion × group [*F*(1, 71) = 3.69, *p* = 0.05]
12				
13			HbO: *F*(1, 61) = 4.02, *p* = 0.04	
14				
15			HbO: *F*(1, 58) = 4.62, *p* = 0.03	
				
17				
18				
19				HbO: identity × emotion × group [*F*(1, 63) = 4.41, *p* = 0.03]
				HbD: group × identity [*F*(1, 63) = 3.8, *p* = 0.05]
20				
21				HbD: group × identity [*F*(1, 65) = 3.79, *p* = 0.05]
				Identity × emotion × group [*F*(1, 65) = 4.61, *p* = 0.03]
				
23				HbD: group × identity [*F*(1, 67) = 4.14, *p* = 0.04]
24			HbO: *F*(1, 66) = 4.45, *p* = 0.03	
